# Selection upon Genome Architecture: Conservation of Functional Neighborhoods with Changing Genes

**DOI:** 10.1371/journal.pcbi.1000953

**Published:** 2010-10-07

**Authors:** Fátima Al-Shahrour, Pablo Minguez, Tomás Marqués-Bonet, Elodie Gazave, Arcadi Navarro, Joaquín Dopazo

**Affiliations:** 1 Department of Bioinformatics and Genomics, Centro de Investigación Príncipe Felipe (CIPF), Valencia, Spain; 2 Institut de Biologia Evolutiva, Universitat Pompeu Fabra (UPF) and Consejo Superior de Investigaciones Científicas (CSIC), Barcelona, Spain; 3 Department of Genome Sciences, University of Washington, Seattle, Washington, United States of America; 4 Howard Hughes Medical Institute, University of Washington, Seattle, Washington, United States of America; 5 Population Genomics Node (National Institute for Bioinformatics, INB), Barcelona, Spain; 6 Institució Catalana de Recerca i Estudis Avançats (ICREA), Barcelona, Spain; 7 CIBER de Enfermedades Raras (CIBERER), Valencia, Spain; 8 Functional Genomics Node (National Institute for Bioinformatics, INB), CIPF, Valencia, Spain; University of California Davis, United States of America

## Abstract

An increasing number of evidences show that genes are not distributed randomly across eukaryotic chromosomes, but rather in functional neighborhoods. Nevertheless, the driving force that originated and maintains such neighborhoods is still a matter of controversy. We present the first detailed multispecies cartography of genome regions enriched in genes with related functions and study the evolutionary implications of such clustering. Our results indicate that the chromosomes of higher eukaryotic genomes contain up to 12% of genes arranged in functional neighborhoods, with a high level of gene co-expression, which are consistently distributed in phylogenies. Unexpectedly, neighborhoods with homologous functions are formed by different (non-orthologous) genes in different species. Actually, instead of being conserved, functional neighborhoods present a higher degree of synteny breaks than the genome average. This scenario is compatible with the existence of selective pressures optimizing the coordinated transcription of blocks of functionally related genes. If these neighborhoods were broken by chromosomal rearrangements, selection would favor further rearrangements reconstructing other neighborhoods of similar function. The picture arising from this study is a dynamic genomic landscape with a high level of functional organization.

## Author Summary

We describe here the most extensive functional cartography of the genomes of multiple species carried out to date. Our study shows, for the first time, how neighborhoods of functionally related genes arise and how they are maintained through evolution following a pattern that is fully consistent with the evolutionary trees of the analyzed species. Contrary to what would be expected, such neighborhoods are not composed of the same genes in different species but rather by genes unrelated, annotated, however, with the same function. Our analysis also reveals that such neighborhoods are dynamically rebuilt in a way that, while the particular genes often change, it is the function of the genes present in the neighborhood, as the ultimate target of selection, that is preserved.

## Introduction

Gene activity, in terms of both intensity [1] and coexpression [2]–[5], does not occur randomly across eukaryotic chromosomes, but in many cases it clusters in certain genomic regions. Nevertheless, the driving force that originated and maintains co-expression neighborhoods is still a matter of controversy [3], [4], [6]–[10]. Several hypotheses have been put forward in order to explain the co-expression of neighboring genes which include the selection for co-regulation of genes with similar functional roles [9], [11], the reduction of gene expression noise in co-localized (but not necessarily functionally related) genes [6], [12] or the formation of clusters of paralogous genes with related functions and expression patterns by tandem duplication [2], [4], [13]. Co-regulation seems to be behind a significant part of the observed coexpression [14],[15] and other features, such as protein interactions seem also be correlated to coexpression [16]–[18]. The emerging portrait from different studies suggests that coexpression in clusters of genes might have both a functional and a neutral (non-functional) component [19].

In order to understand the real extent of this phenomenon we have produced a detailed functional cartography of the genomes of eight eukaryotic model species: *Homo sapiens*, *Pan troglodytes*, *Mus musculus*, *Rattus norvegicus*, *Gallus gallus*, *Danio rerio*, *Drosophila melanogaster*, *Caenorhabditis elegans*, and *Arabidopsis thaliana*. A sliding window (see Materials and Methods section) was moved along all chromosomes and the enrichment in Gene Ontology [20] (GO) functional terms within each window was analyzed [21].

## Results

### Functional neighborhoods in eukaryotic genomes

One of the most remarkable results of our analysis is the rich functional landscape that it unveils. When the distribution of the functional annotations of the genes is analyzed by a sliding window (see Materials and Methods) it becomes apparent that genomes are formed by a large amount of functional neighborhoods. These range in the well annotated species from a 3% (*Arabidopsis thaliana*) to a 12% (*Mus musculus*) of the genes (Table 1). For example, in *Homo sapiens*, chromosomes 11 and 19 show a high number of genes in functional clusters (17.6% and 25.6%, respectively), which agrees with previous observations about the special properties of these chromosomes [22]. In mammals, sex chromosomes present a significant deviation in percentages when compared to autosomes (e.g. below 2% in X chromosomes or an extreme value of 35% in the Y chromosome of *Mus musculus*). See also Figure S1 that depicts the distribution of functional neighborhoods across the chromosomes of the species studied and Figure S2 with more details on the functions found in the neighborhoods. Table S3 list the genes contained in the functional neighborhoods found. Differences between human and chimpanzee in the mean gene density and percentage of genes in functional neighborhoods seem to be greater than it might be expected from their phylogenetic proximity. However, the considerable amount of chromosomal rearrangements between the genomes of humans and chimpanzees, most of which happened in the chimpanzee lineage [23] and were caused by repetitive elements [24] and lineage-specific segmental duplications [25], can provide an explanation for the observed differences. These differences strongly suggest the existence of selective pressures acting differentially on the respective functional neighborhoods. Our results are also in agreement with indirect evidences from inbred strains of mice based on linkage disequilibrium, which indicate that a quarter or more of the mammalian genome could consist of chromosome regions containing clusters of functionally related genes [26].

**Table 1:** Characteristics of functional neighborhoods.

	*Homo sapiens*	*Pan Troglodytes*	*Mus musculus*	*Rattus norvegicus*	*Gallus gallus* 1	*Danio rerio* 1	*Drosophila melanogaster*	*Caenorhabditis elegans*	*Arabidopsis thaliana*
Number of functional neighborhoods	265	208	315	267	25	55	146	163	193
Percentage of genes in functional neighborhoods^2^	7.2%	4.71%	11.9%	12.8%	1.0%	1.4%	5.3%	5.8%	3.0%
Mean GC content (p-value)	42.6% (<10^−30^)	41.4% (0.0352)	42.29% (0.0019)	42.72% (<10^−30^)	41.55% (NS)	36.37% (NS)	42.39% (NS)	35.8% (0.0015)	35.66% (NS)
Mean gene density in functional neighborhoods^3^ (p-value)	85.84 (<10^−30^)	57.35 (<10^−30^)	70.77 (<10^−30^)	70.26 (<10^−30^)	69.32 (0.0154)	61.96 (0.0014)	54.85 (0.0061)	63.59 (<10^−30^)	52.07 (NS)
p-value of K-S test of co-expression in functional neighborhoods	7×10^−19^	2×10^−29^	7×10^−11^	1.2×10^−8^	NA	2.8×10^−15^	0.01	1.3×10^−17^	1.5×10^−5^

Functional neighborhoods display both a higher GC content and mean gene density which has been described as characteristic of tightly regulated chromosomal domains (28).

1 These species are seriously affected by a poor annotation of the genes.

2 Only genes annotated with significantly clustered GO terms are considered here. Genes within the limits of a functional neighborhood that do not match the significant GO term are not considered as members of the cluster.

3 Total gene density in the functional neighborhoods is reported, including all genes within the limits of the neighborhood independently of the GO terms associated to them. Window size was selected to include, approximately, 50 genes per window with slight variations among organisms.

### Functional neighborhoods are conserved across the phylogeny

**Figure 1:**
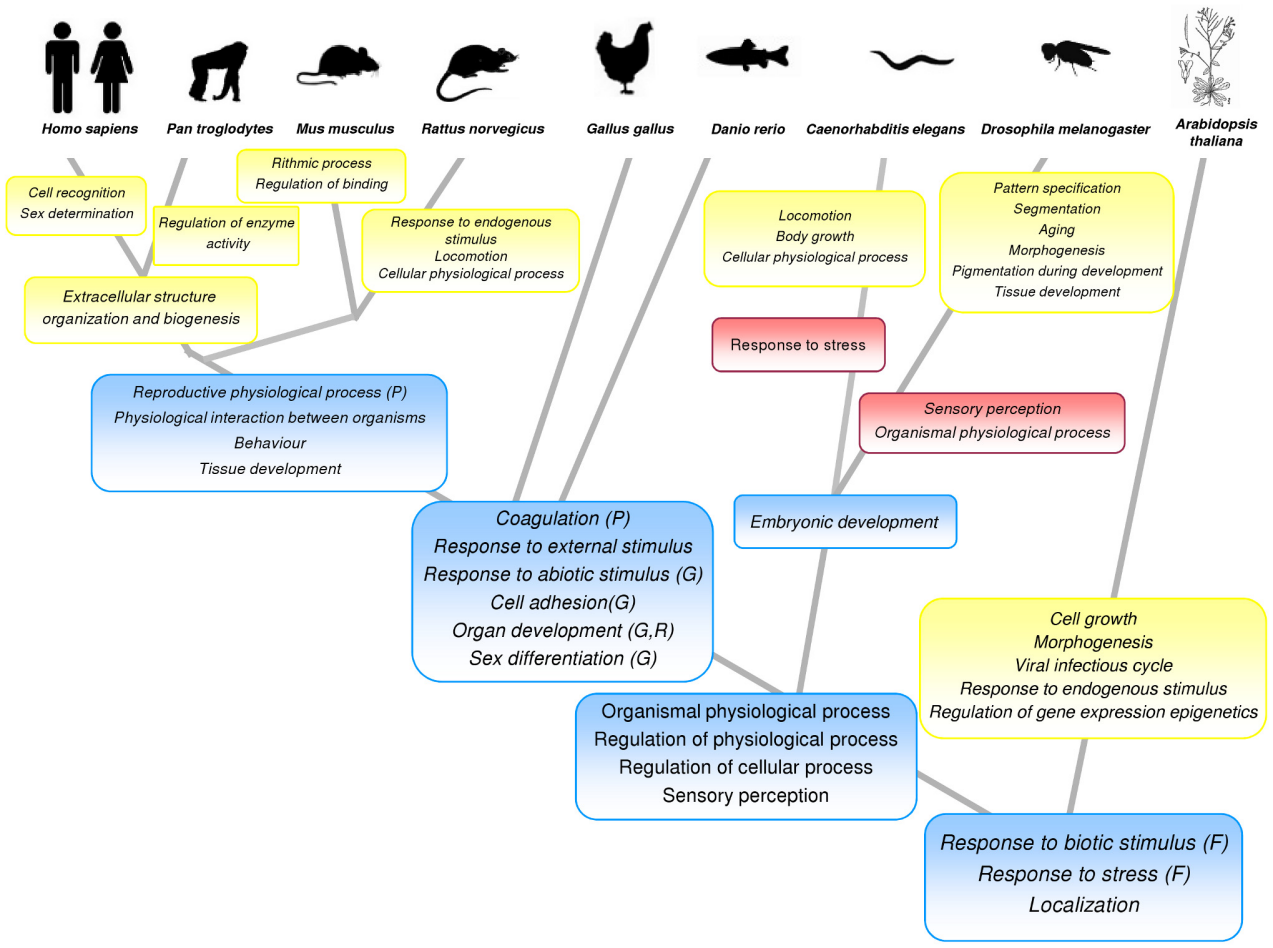
Distribution of functions present in functional neighborhoods along the phylogeny. The point at which a function makes up a functional neighborhood has been deduced from the species sharing functional clusters with this particular GO term. Boxes in yellow contain GO terms unique to taxa, boxes in blue contain GO terms common to clades and boxes in pink contain GO terms lost in these lineages. In the figure, terms labeled with **P** were not found in ape, with **G**: were not found in chicken, with **R** were not found in rat and with **F** were not found in fish.

Some of the functional categories found in functional neighborhoods are unique, and, as previously suggested [9], probably account for lineage or species-specific characteristics. Nevertheless, many GO term clusters were consistently shared by different species. When the GO terms found within the functional clusters are mapped over the eukaryotic phylogeny the distribution across species of the vast majority of them is fully compatible with the tree topology. Table 2 shows how different functional modules are distributed across species, arranged according their relative positions in the phylogeny. Figure 1 shows the most parsimonious phylogenetic positions of the functions consistently found in neighborhoods. Most trends are clear, despite some discrepancies in *G. gallus* or *D. rerio*, probably due to the preliminary stage of the annotations in these organisms. Thus, for example, *Response to biotic stimulus*, *Response to stress* and *Localization* seem to define functional neighborhoods common to all the eukaryotes. Actually, clustering of stress-related genes was described to occur during evolution of the *S. cerevisiae* genome [27]. Other terms, such as *Organismal physiological process*, *Regulation of physiological process*, *Regulation of cellular process* and *Sensory perception*, are characteristic to all animals. In plants (at least in its unique representative, *A. thaliana*) we found different terms, such as *Cell growth*, *Viral infectious cycle*, *Regulation of gene expression epigenetics*, as apomorphisms. Shared by all vertebrates are GO terms such as *Coagulation*, *Response to external stimulus*, *Response to abiotic stimulus*, *Cell adhesion*, *Organ development* and *Sex differentiation* (with the exception of chicken, as already mentioned). Invertebrates share clusters with the GO term *Embryonic development*. Finally, mammals share functional neighborhoods with GO terms such as *Reproductive physiological process*, *Physiological interaction between organism* and *Behaviour*, most of them making reference to more complex, social or interactive behaviors displayed by these animals. Human and chimpanzee are the closest species and share almost all the GO terms in functional neighborhoods.

**Table 2:** Number of genes in functional neighborhoods.

	*Homo sapiens*			*Pan troglodytes*			*Mus musculus*			*Rattus norvergicus*			*Gallus gallus*			*Danio rerio*			*Caenorhabditis elegans*			*Drosophila melanogaster*		
	genes	%	Orth.	genes	%	Orth.	genes	%	Orth.	genes	%	Orth.	genes	%	Orth.	genes	%	Orth.	genes	%	Orth.	genes	%	Orth.
organismal physiological process	1885	15.23	-	1673	10.82	69.61	1042	11.23	31.62	604	20.20	21.31	120	4.17	0	164	16.46	0	156	35.90	0	475	0	0
regulation of physiological process	2917	8.12	-	2671	4.34	65.52	2318	1.77	31.71	1240	1.94	45.83	432	0	0	718	3.48	0	650	14.31	0	903	3.77	2.94
regulation of cellular process	2980	7.89	-	2765	4.30	67.23	2367	1.73	31.71	1247	1.92	45.83	437	0	0	728	5.08	27.03	607	16.47	0	932	2.58	4.17
sensory perception	603	35.66	-	455	29.45	60.45	357	36.96	37.89	125	37.60	27.66	26	19.23	20.00	66	37.88	0	90	61.11	0	194	5.67	0
coagulation	85	3.53	-	57	0	0	50	6.00	100	34	17.65	0	11	54.55	0	21	0	0						
response to cexternal stimulus	464	6.68	-	433	6.93	90	311	6.43	70	140	11.43	56.25	32	15.62	0	48	10.42	0						
response to abiotic stimulus	384	9.64	-	414	5.31	100	280	9.29	50	148	8.78	61.54	27	0	0	75	13.33	0						
cell adhesion	558	5.73	-	431	4.18	100	428	10.98	72.34	251	13.94	54.29	94	0	0	85	20	29.41						
organ development	524	2.48	-	798	0	0	752	0.93	0	157	0	0	45	0	0	157	3.82	0						
sex differentiation	40	7.50	-	62	4.84	100	46	6.52	100	15	13.33	100	1	0	0	5	80	50						
reproductive physiological process	50	18.97	-	83	0	0	39	10.26	75	12	25	66.67												
physiological interaction between organisms	52	21.15	-	56	8.93	100	16	25	75	6	50	66.67												
behavior	185	18.92	-	250	12.40	83.87	177	9.60	74.67	58	20.69	66.67												
Average		12.42	-		9.15			10.52			18.54			23.39			21.16			31.95			4.01	

The most left column correspond to the GO terms defining functional neighborhoods. The rest of columns correspond to the analyzed species. Each species' column is divided into three sub-columns labeled as: 1) “genes”, which correspond to the total number of genes in the genome of this particular species annotated with the GO situated in the first column of the corresponding row, 2) “%”, which corresponds to the percentage of these genes found within a functional neighborhood and 3) “Orth.”, which corresponds to the percentage of the genes within the functional neighborhood which are orthologous with respect to their human counterparts.

### Relationship between gene coexpression and common annotations in functional neighborhoods

Our results support a strong causal relationship between local coexpression and local co-functionality. For each GO term, the correlations among gene expression profiles of genes located within the functional neighborhood were compared to the corresponding correlations among the rest of genes belonging to the same GO term located outside the neighborhood (see Materials and Methods section). Table 1 shows that there is a significantly higher degree of coexpression in genes belonging to a given functional class when they are packed within a functional neighborhood than when they are elsewhere in the genome. This result, along with the lack of a significant relative enrichment of tandem duplications (see below), points out to coexpression as the most plausible driving force for the existence of functional neighborhoods.

### Functional neighborhoods do not mainly result from duplication

If functional neighborhoods are originated as a simple result of tandem duplications of genes, different copies may or may not acquire different functions, but, in any case, they might share GO terms. A simple analysis of the number of paralogous contained in the regions shows that the percentages range from 14% (human) to 28% (rat), which corresponds almost exactly to the percentage of paralogous in the corresponding genomes. The number of paralogous among the GO genes is higher (around the 40%) which, again, corresponds to the percentage of paralogous within the GO categories. Given that functional annotations could be transferred by similarity [20], an artifactual accumulation of identical GO terms could be observed in this scenario. To discard this possibility we performed two different analyses. Firstly, we examined whether functional neighborhoods are enriched with segmental duplications. For every species for which appropriate information is available (see Materials and Methods section), a binomial test was used to determine if the number of segmental duplications within functional clusters is larger than what be expected according to their size. Our results allow rejecting the hypothesis that recent segmental duplications are contributing to the formation of functional clusters (Table 3: p-values always n.s.). Still, the possibility remains that the clusters we observe are the result of ancient duplication events that have diverged too much to be detected as such. In that scenario, different paralogous copies of a gene may still be similar at the protein level and form functionally related gene families. Our second analysis accounts for the effect of ancient duplications by examining the distributions of the average number of BLASTP [28] hits within the regions containing functional clusters and comparing them to the genome-wide background (that is the rest of equivalent chromosomal regions). When compared, both distributions of BLASTP hits were indistinguishable for all the studied organisms (See Figure S3 and Table S1). Thus, our general multispecies analysis demonstrates that the emergence of new genes by tandem duplications cannot be the general explanation behind the origin of functional neighborhoods.

**Table 3:** Segmental Duplication (SD) analysis.

Species	Number of SDs in functional neighborhoods	Number of SDs in the rest of the genome	Total size (in Mbps) of functional neighborhoods	Total size (in Mbps) rest of the genome	Observed proportion of SDs in functional neighborhoods	Expected proportion of SDs in functional neighborhoods	Observed proportion of SDs in rest of the genome	Expected proportion of SDs in rest of the genome	Total genome size in Mbps (golden path)	P-value
**Human**	1630	3795	932.50	1957.03	0.3004	0.3227	0.6995	0.6773	2889.53	n.s.
**Mouse**	1851	3399	952.35	1628.47	0.3526	0.3690	0.6474	0.6310	2580.82	n.s.
**Chicken**	602	13366	70.00	983.97	0.0431	0.0664	0.9569	0.9336	1053.97	n.s.

### Functional neighborhoods shared by clades are not composed of ortholog genes

Surprisingly, the genes found in functional neighborhoods shared by different organisms are not necessarily orthologous (see Table 2). That is, when two species share functional neighborhoods, the genes forming these clusters may be different in each species. One might expect that if such functional neighborhoods emerged in a particular period of the evolution and apparently were maintained since then (given that they are shared by all the descendant species), these clusters were essentially composed by ortholog genes. Nevertheless, this is not the case.

Table 2 shows three columns for each organism. The first one is the number of genes annotated with the GO functional categories shared by the different relevant clades (mammalians, vertebrates and animals), the second one is the proportion of such genes that were found in functional clusters where this particular GO category was significantly over-represented and the third column is the proportion of such genes with a human ortholog. The two most important observations that can be made from Table 2 are: i) the proportion of genes in functional neighborhoods in each functional category tend to be approximately constant across taxa (with a few exceptions). For example, a large proportion of genes belonging to *sensory perception* cluster in neighborhoods (over 20%, except in the case of *Drosophila*) across the species in Table 2 while behavior genes keep their proportions approximately between 10 and 20%. The results found in *Gallus gallus* and *Danio rerio* are less conclusive probably because of the preliminary of the functional annotation. And ii) the genes found in the shared functional neighborhoods in different organisms do not have a relationship of orthology. That is, the proportion of ortholog genes with respect to their human counterparts is significantly lower than expected from an evolutionary event in which groups of functionally related genes gathered in the genome and were subsequently maintained along evolution. The presence in the functional clusters of mammals of a significantly high number of repetitive elements (SINE), which are known to be involved in rearrangement processes [29], [30], suggest that such regions may be undergoing a continuous process of rearrangement and selection is ultimately favoring the presence of genes belonging to the functional categories required by the organisms. In fact we observed a significant enrichment in SINEs in the functional regions of human (p<0.0001), mouse (p = 0.0057) and rat (p = 0.0002). From this point of view, a number of functional categories would require to have a minimum number of genes clustered together in the genome for optimal transcriptional activity, but not necessarily the same set of ortholog genes. Our findings actually suggest that it is the fraction of genes of a given function, and not the particular genes, which is relevant from the point of view of the transcriptional efficiency. This is in agreement with previous suggestions of other authors about the existence of a functional component reflected in the physical proximity of the genes that would be favoring their simultaneous co-expression [3], [4], [7]. This observation is also compatible with a dynamic scenario in which function, understood as a system of genes at work, rather than a particular static set of orthologous genes, is the target of natural selection [31].

### Functional neighborhoods shared between species are significantly enriched with breaks of synteny

The comparative study of synteny conservation can throw some light on a scenario in which phylogenetically consistent functional neighborhoods composed by non orthologous genes occur. Synteny data are available for a number of species, but since the highest quality information has been obtained for the human-chimpanzee synteny relations [23], [32], [33], we have focused in functional neighborhoods shared between these two species. Humans and chimpanzees are separated by 10 major chromosomal rearrangements [34] and many small ones that imply many breaks of synteny between the two species. We observed that, as an average, functional neighborhoods shared between these species are significantly enriched with such breaks of synteny (Table 4). This is another surprising result: not only functional clusters are not particularly conserved, but they seem to be highly reorganized. These clusters appear to be enriched with rearrangement breakpoints relative to the rest of the genome (for example, using the synteny information from the Newman et al. Dataset [23] this means ∼0.15 Breakpoints/Mb in neighborhoods vs. ∼0.09 Bkp/Mb in the rest of the genome, Chi-square test, p-value = 4.2×10^−6^, see Table 4). This renders further support to the idea that there are strong selective pressures that maintain a minim number of genes with certain functions within clusters and is consistent with the observation reported above of clusters shared between species that, in spite of having the same functions, do not share the same ortholog genes.

**Table 4:** Functional neighborhoods shared between humans and chimpanzees.

Data from Newman et al (2005)								
			OBSERVED			EXPECTED		
	Length (Mbp)	% of total lenght	BoS	% of total BoS	BoS density * Mb	BoS	Chi-square value	P-value (Chi-Square)
**Shared Neighborhoods**	754.72	0.25	118	0.35	0.1563	82		
**Rest of the genome (including not shared neighborhoods)**	2325.70	0.75	216	0.65	0.0929	252		
**Total**	3080.42		334			334	21.17	4.2×10^−6^

Data from Feuk et al (2005) (Validated Inversions>25 Kb)								
			OBSERVED			EXPECTED		
	Length (Mbp)	% of total lenght	BoS	%of total BoS	BoS density * Mb	BoS	Chi-square value	P-value (Chi-Square)
**Shared Neighborhoods**	754.72	0.25	42	0.32	0.0556	32		
**Rest of the genome (including not shared neighborhoods)**	2,325.70	0.75	89	0.68	0.0382	99		
**Total**	3,080.42		131			131	4.05	0.0442

Density of breaks of synteny (BoS) in these neighborhoods *vs.* the rest of the genome. The density of breaks of synteny is higher in shared neighborhoods.

Moreover, when functional neighborhoods are classified according to the percentage of orthologous genes they contain, highly orthologous neighborhoods present significantly less synteny breaks than low-orthology neighborhoods (∼0.1 vs. ∼0.2 Bkps/Mb, p-value = 0.000231 in the Newman's Dataset [23]) and a synteny conservation that is similar, or even stronger, than the genome average. The situation is the opposite in low orthology neighborhoods (see Table 5 and Table 3). In both cases functional neighborhoods present an internal level of coexpression that is significantly higher than the level observed in genes belonging to the same functional categories when dispersed across the genome (high orthology p-value = 1.197×10^−21^ low orthology p-value = 1.696×10^−17^; see also Table 1), defining in this way functional, coexpression neighborhoods. The fact that the degree of coexpression is lower in low orthology neighborhoods than in the case of high orthology ones would be compatible with a dynamic scenario of continuous reconstitution of low orthology domains where the expression process was not fully optimized yet. Without entering in the detail on where the conservation of the neighborhoods came from, the observation that genes with altered neighborhood are more likely to undergo expression divergence than genes with conserved neighborhood was already made [35]. This scenario has also some similitude to the one proposed by Poyatos and Hurst for yeast [16], in which selection for high levels of co-expression would correlate with high levels of recombination rates, which, in turn mean high levels of chromosomal rearrangement and increase the probability of breakage of the co-expressed cluster. It has also been observed that co-expression between adjacent genes is positively correlated with the probability that those genes would be apart in the genome of a different species [36]. The fact that, in our case, highly orthologous clusters present the highest co-expression levels and lowest rearrangement rates suggests, however, a different cycle: cluster would contain many rearrangement breakpoints because natural selection would favor the reconstruction of clusters via chromosomal reorganization. In addition, this is consistent with the fact that the rearrangement breakpoints tend to reduce and not to increase recombination while segregating in a population [37].

**Table 5:** Functional neighborhoods shared between humans and chimpanzees.

Data from Newman et al (2005)								
			OBSERVED			EXPECTED		
Neighborhoods	Length (Mbp)	% of total lenght	BoS	% of total BoS	BoS density*Mb	BoS	Chi-square value	P-value (Chi-Square)
**Neighborhoods< median orthology**	383.02	0.51	80	0.68	0.2089	60		
**Neighborhoods> median orthology**	371.70	0.49	38	0.32	0.1022	58		
**Total**	754.72		118			117	13.56	2.31×10^−4^

Data from Feuk et al (2005) (Validated Inversions >25 Kb)								
			OBSERVED			EXPECTED		
Clusters	Length (Mbp)	% of total lenght	BoS	% of total BoS	BoS density*Mb	BoS	Chi-square value	P-value (Chi-Square)
**Neighborhoods< median orthology**	383.02	0.51	36	0.86	0.0939	21		
**Neighborhoods> median orthology**	371.70	0.49	6	0.14	0.0161	21		
**Total**	754.72		42			42	20.54	5.83×10^−6^

Density of breaks of synteny (BoS) in neighborhoods with high orthology *vs.* clusters with low orthology. Highly orthologous clusters present lower density of synteny breaks.

## Discussion

Results presented here demonstrate that a large fraction of the genome is arranged in neighborhoods of functionally related genes that are not the result of tandem duplications but of reorganization. Coexpression has systematically observed to occur within functionally related genes defining the functional neighborhoods. The fact that functions shared across species analyzed is compatible with the evolutionary pattern of speciation constitutes strong evidence in favor of the existence of a selective force that produced and maintained the observed functional neighborhoods, even if different sets of genes make them up in different species. Moreover, in an apparent paradox, functional neighborhoods, which in one hand are conserved across evolution, appear to be enriched with rearrangement breakpoints when compared to the rest of the genome. Both observations suggest that selection is operating at the level of functional neighborhoods, no matter their particular genic composition. In this scenario, when a functional neighborhood is broken by a chromosomal rearrangement, selection would favor new rearrangements that tended to reconstitute a neighborhood with the same function, although the gene composition may differ from the ancestral one.

Actually, the number of functional neighborhoods found constitutes, most probably, an underestimation of its real number because of two facts: i) the testing scheme used is conservative and ii) this study considers only neighborhoods collinear in the chromosomes but no spatial neighborhoods formed by the tridimensional conformation of the nucleus. The real spatial conformation of the nucleus is still unknown but new data are continuously arising [38], [39] and the relationship of physical proximity with gene expression [40] and their possible functional implications [41] are becoming increasingly clear. As new information is available this extreme will be studied in more detail.

## Materials and Methods

### Data

The genomes of *Homo sapiens*, *Mus musculus*, *Pan troglodytes*, *Rattus norvegicus*, *Gallus gallus*, *Danio rerio*, *Drosophila melanogaster*, *Caenorhabditis elegans*, were taken from Ensembl [42] and the genome of *Arabidopsis thaliana* was obtained from AtEnsembl, (release 29, http://atensembl.arabidopsis.info). All the microarrays were chosen to represent conditions as normal and as non-pathological as possible. The following datasets, taken from the ArrayExpress database (http://www.ebi.ac.uk/microarray-as/ae/), were used: Human: E-AFMX-5; Mouse: E-AFMX-4; Fish: E-TABM-33; Fly E-MEXP-127, E-MEXP-152, E-MEXP-202, E-MEXP-493, E-MEXP-88; Worm:E-SMDB-1398, E-SMDB-3540, E-SMDB-3539, E-SMDB-3592 and Plant: E-TABM-17. No comparable data were found for chicken and, thus, it was excluded from the analysis of expression data. More information on the data used and the results obtained can be found at: http://bioinfo.cipf.es/publications/additionaldata/functional_clusters.

### Synteny analysis

The analysis of density of breakpoints in windows of functional enrichments was performed using the breaks of synteny between Humans and Chimpanzees from Newman et al [43] and Feuk et al [32]. For the later, only the set of rearrangements >25Kb was used. The first dataset (Newman's dataset), was built blasting Fosmid pair-end sequences into the human genome, so it does not depend on the quality of the chimpanzee assembly. The second dataset (Feuk's dataset), was constructed comparing the order of genes between assemblies of the two species, and thus, it is likely to be affected by the lower quality of the assembly of the chimpanzee genome available at the time of the publication of the paper by Feuk et al [32].

### Sliding windows approach

All the chromosomes of the studied species were scanned by means of a sliding window. In order to be compliant with previous studies [1] window size was adjusted in each species to contain, on average, approximately 50 genes (see Table S2). The windows are moved along all chromosomes in steps of half a window. A conventional method of functional enrichment implemented in the FatiGO program [21], which is part of the Babelomics (http://www.babelomics.org) suite for functional analysis [44], [45], was used to study the significant over-representation of GO terms in each window. Briefly, the method builds a 2×2 contingency table for each functional term checked for each window and applies an exact Fisher's test. The p-values obtained for all the windows were FDR-corrected [46] taking into account all the tests conducted in all the organisms. Figure S4 shows a schema of the procedure followed to detect functional neighborhoods.

### Testing for duplication events

Available data on segmental duplications were downloaded from the Eichler Lab databases (http://eichlerlab.gs.washington.edu/database.html). To avoid coordinate translation biases, only species for which the segmental duplications and the gene annotation assemblies were concordant were used. Thus, the segmental duplication analysis involves only human, chimpanzee, chicken and mouse. The proportion of segmental duplications contained in windows containing functional neighborhoods was compared to the proportion of segmental duplications in windows without clusters (i.e. in the rest of the genome) after removing from analysis ambiguously located segmental duplications. A binomial test was used to determine whether the number of segmental duplications inside windows with functional clusters was larger than expected under the null hypothesis of random distribution of segmental duplications with respect to functional neighborhoods.

A further way to infer the number of recent and ancient duplication events in a window is using the number of BLASTP [28] hits that any of the genes contained in it produces when searched against a all the genes within the region. A region including only single-copy genes not belonging to a gene family and no ancient or new tandem duplications will theoretically produce only one hit per gene (the gene against itself). A region consisting of a group of genes amplified N times will produce N BLASTP hits per gene. Situations in between these extremes will produce more than one BLASTP hit for some genes. For each window we constructed a BLASTP database with the corresponding proteins. Then, all the proteins corresponding to the genes in the window were blasted (using BLASTP) against the corresponding database, and the total number of BLASTP hits with a percentage of similarity over a threshold of ***T%*** was normalized and recorded. Values of 98% and 95% were used as thresholds. Again, for each organism, windows containing functional neighborhoods were compared to a background consisting of the rest of windows without significant functional neighborhoods inside.

### Coexpression analysis

For each GO term the pairwise Pearson correlations among genes located within the functional neighborhood is compared to the corresponding correlations among the rest of genes not located in the neighborhood, by means of a Kolmogorov-Smirnov test.

## Supporting Information

Figure S1 Multi-species cartography of genomes enriched in genes with related functions. Functional neighborhoods are represented by arrows at their corresponding chromosomal coordinates. See text for the versions of the databases used for the coordinate mapping. The species analyzed appear in the pages below and are: a) *Homo sapiens* b) *Pan troglodytes* c) *Mus musculus* d) *Rattus norvegicus* e) *Gallus gallus* f) *Danio rerio* g) *Drosophila melanogaster* h) *Caernohabditis elegans* i) *Arabidopsis Thaliana*.
(0.08 MB DOC)

Figure S2 Distribution of significant GO biological processes terms present in functional neighborhoods in the different genomes analyzed.
(0.11 MB DOC)

Figure S3 Distribution of BLASTP hits with an identity over the 98% and 95% in the different genomes studied for the functional neighborhoods (red) and for the rest of the genome (black).
(0.57 MB DOC)

Figure S4 Schema of the procedure followed. See Materials and Methods section.
(0.20 MB DOC)

Table S1 Duplication events t-test comparing the distribution of BLASTP hits for functional neighborhoods versus BLASTP hits for the rest of the genome.
(0.04 MB DOC)

Table S2 Sliding window sizes used for scanning all the chromosomes of the studied species. Window size was adjusted in each species to contain, on average, approximately 50 genes.
(0.04 MB DOC)

Table S3 List of the genes contained in the functional neighborhoods found.
(2.06 MB XLS)
