# Supplementary material for: Selection upon Genome Architecture: Conservation of Functional Neighborhoods with Changing Genes
Source: PLoS Comput Biol. 2010 Oct 7;6(10):e1000953. doi: 10.1371/journal.pcbi.1000953 (PMC2951340; doi:10.1371/journal.pcbi.1000953)
Supplement: Figure S1 — Multi-species cartography of genomes enriched in genes with related functions. Functional neighborhoods are represented by arrows at their corresponding chromosomal coordinates. See text for the versions of the databases used for the coordinate mapping. The species analyzed appear in the pages below and are: a) Homo sapiens b) Pan troglodytes c) Mus musculus d) Rattus norvegicus e) Gallus gallus f) Danio rerio g) Drosophila melanogaster h) Caernohabditis elegans i) Arabidopsis Thaliana. (0.08 MB DOC) [file pcbi.1000953.s001.doc]

**Supplementary information**

**Selection upon genome architecture: conservation of functional neighborhoods with changing genes**

## Fátima Al-Shahrour, Pablo Minguez, Tomás Marqués-Bonet, Elodie Gazave, Arcadi Navarro and Joaquín Dopazo

**Figure S1**. Multi-species cartography of genomes enriched in genes with related functions. Functional neighborhoods are represented by arrows at their corresponding chromosomal coordinates. See text for the versions of the databases used for the coordinate mapping. The species analyzed appear in the pages below and are: a) *Homo sapiens* b) *Pan troglodytes* c) *Mus musculus* d) *Rattus norvegicus* e) *Gallus gallus* f) *Danio rerio* g) *Drosophila melanogaster* h) *Caernohabditis elegans* i) *Arabidopsis Thaliana*.


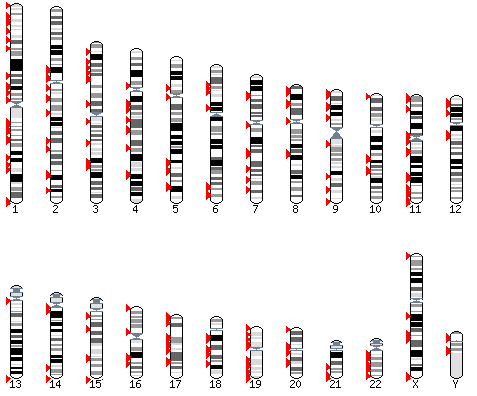


1. *Homo sapiens*


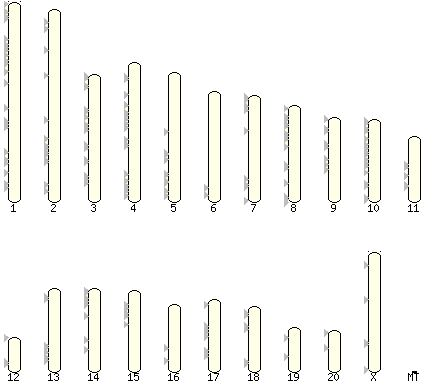


b)*Pan troglodytes*


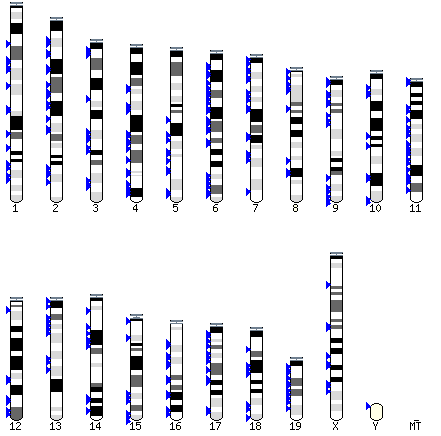


c) *Mus musculus*


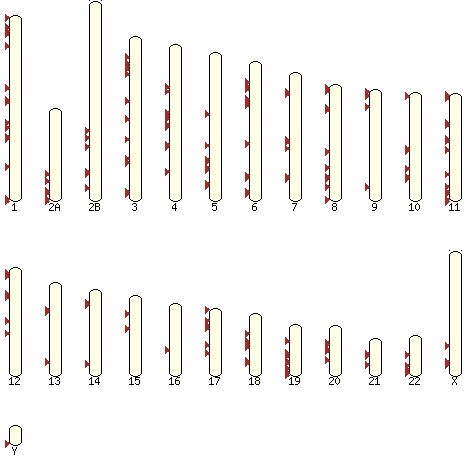


d) *Rattus norvegicus*


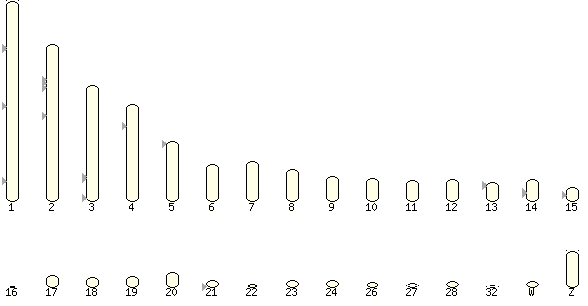


e) *Gallus gallus*


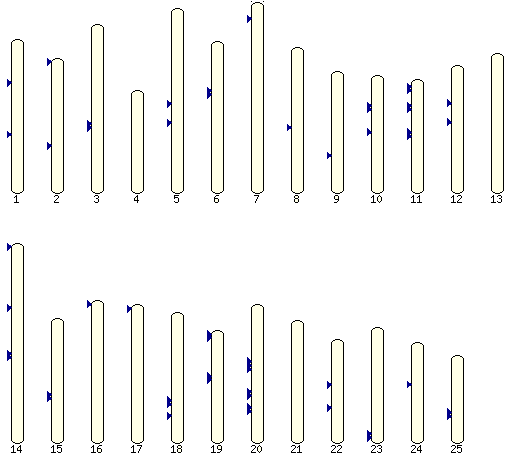


f) *Danio rerio*


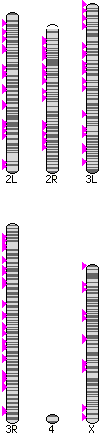

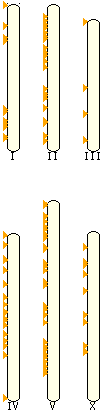

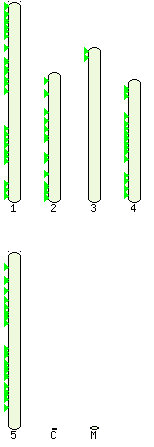


g) *Drosophila melanogaster* h) *Caernohabditis elegans* i) *Arabidopsis thaliana*.
