# Supplementary material for: Selection upon Genome Architecture: Conservation of Functional Neighborhoods with Changing Genes
Source: PLoS Comput Biol. 2010 Oct 7;6(10):e1000953. doi: 10.1371/journal.pcbi.1000953 (PMC2951340; doi:10.1371/journal.pcbi.1000953)
Supplement: Figure S2 — Distribution of significant GO biological processes terms present in functional neighborhoods in the different genomes analyzed. (0.11 MB DOC) [file pcbi.1000953.s002.doc]

**Supplementary information**

**Selection upon genome architecture: conservation of functional neighborhoods with changing genes**

## Fátima Al-Shahrour, Pablo Minguez, Tomás Marqués-Bonet, Elodie Gazave, Arcadi Navarro and Joaquín Dopazo

**Figure S2:** Distribution of significant GO biological processes terms present in functional neighborhoods in the different genomes analyzed.
