# Supplementary material for: Selection upon Genome Architecture: Conservation of Functional Neighborhoods with Changing Genes
Source: PLoS Comput Biol. 2010 Oct 7;6(10):e1000953. doi: 10.1371/journal.pcbi.1000953 (PMC2951340; doi:10.1371/journal.pcbi.1000953)

**Supplementary information**

**Selection upon genome architecture: conservation of functional neighborhoods with changing genes**

## Fátima Al-Shahrour, Pablo Minguez, Tomás Marqués-Bonet, Elodie Gazave, Arcadi Navarro and Joaquín Dopazo

**Figure S3** Distribution of BLASTP hits with an identity over the 98% and 95% in the different genomes studied for the functional neighborhoods (red) and for the rest of the genome (black).


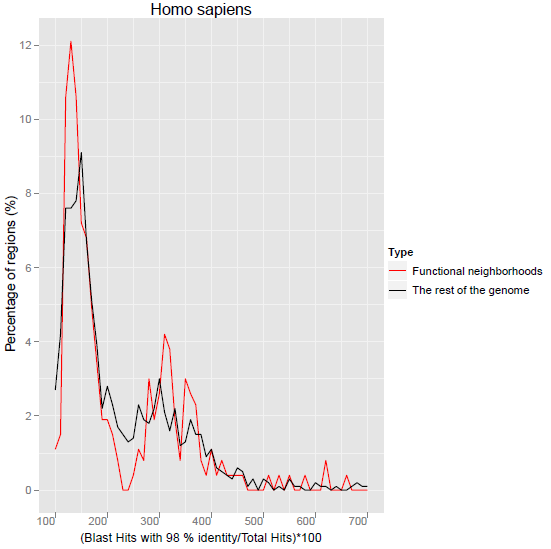


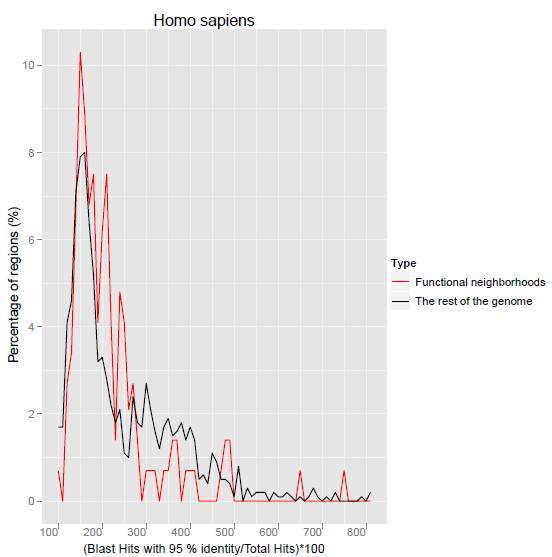


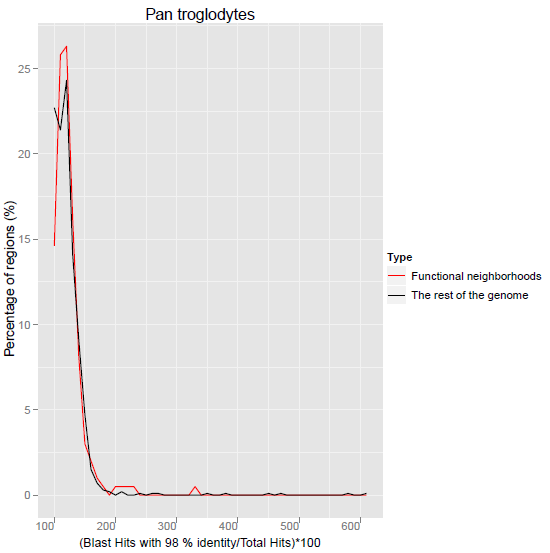


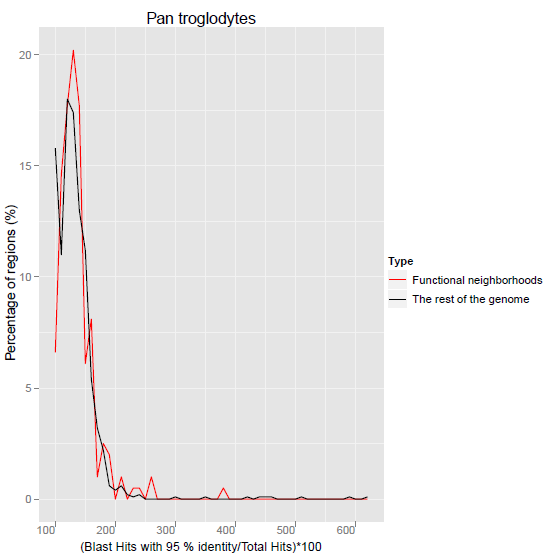


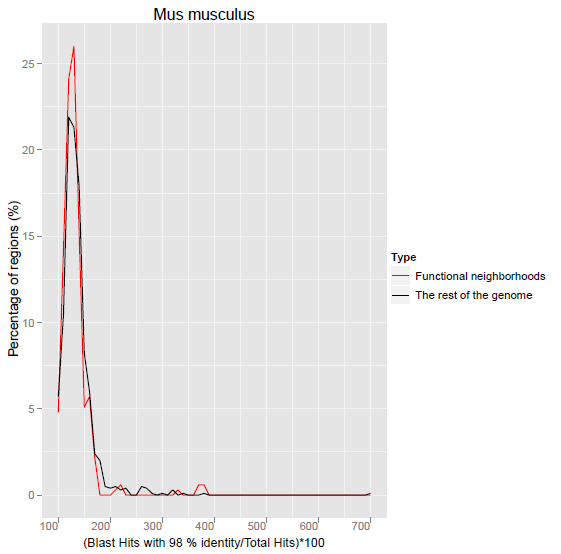


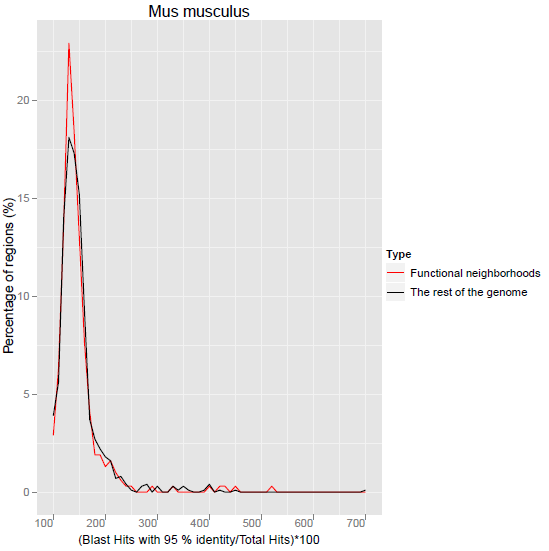


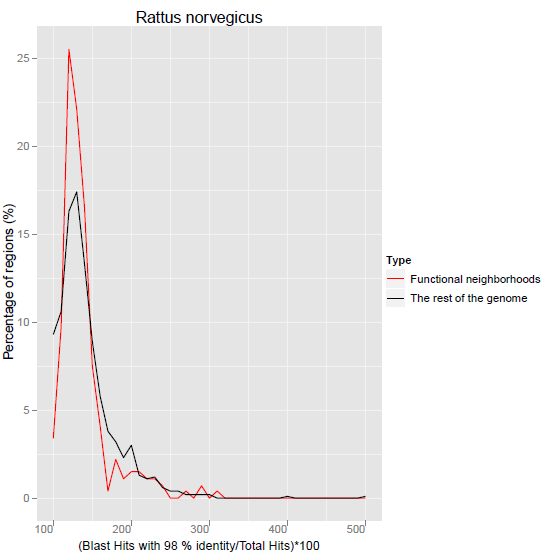


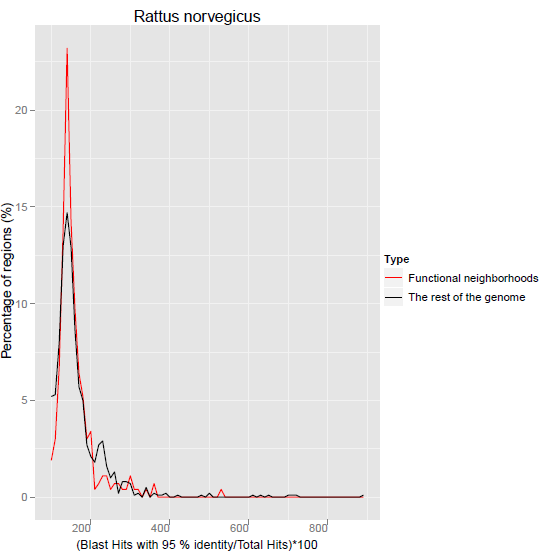


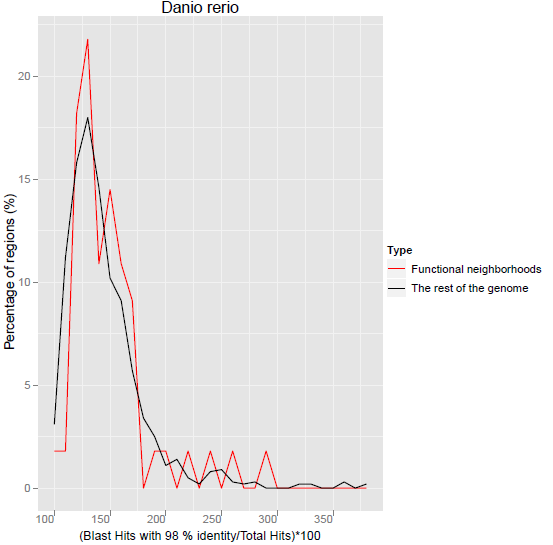


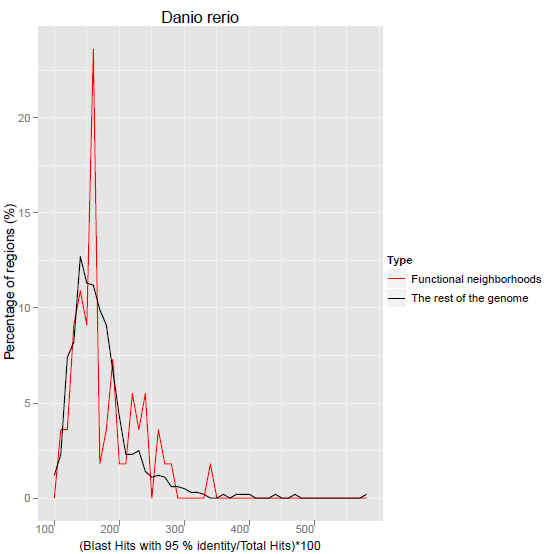


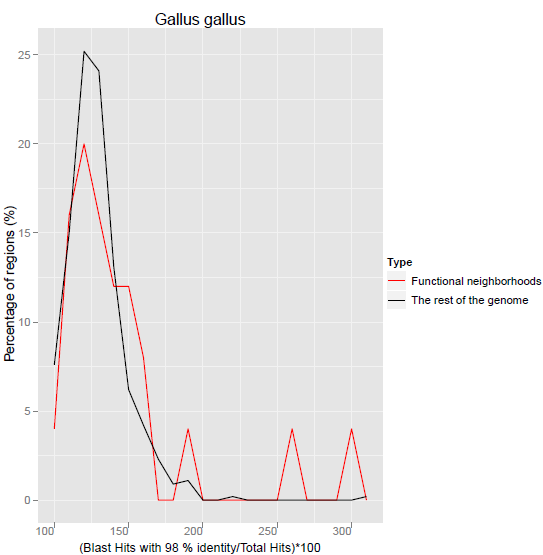


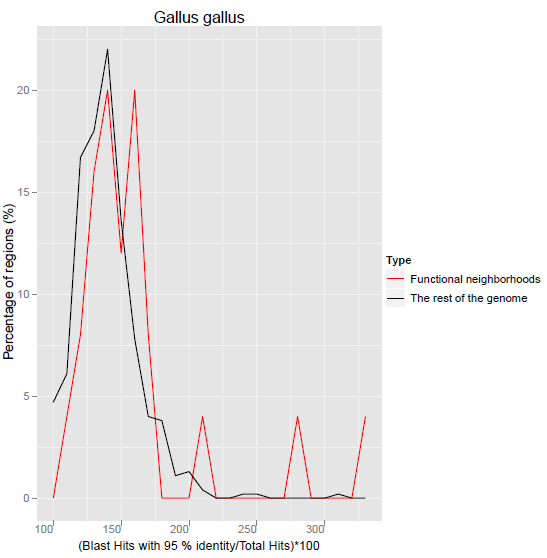


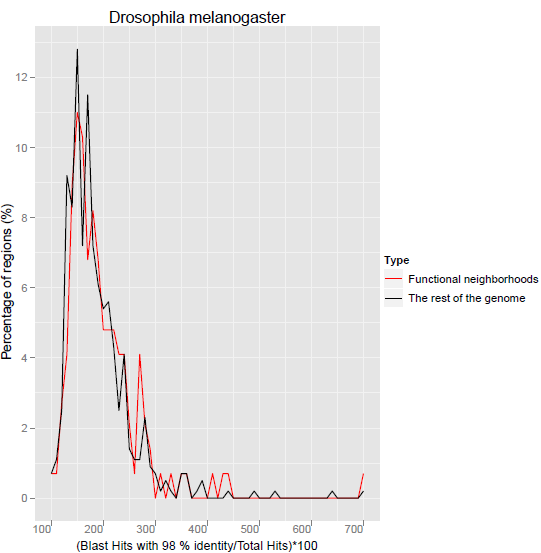


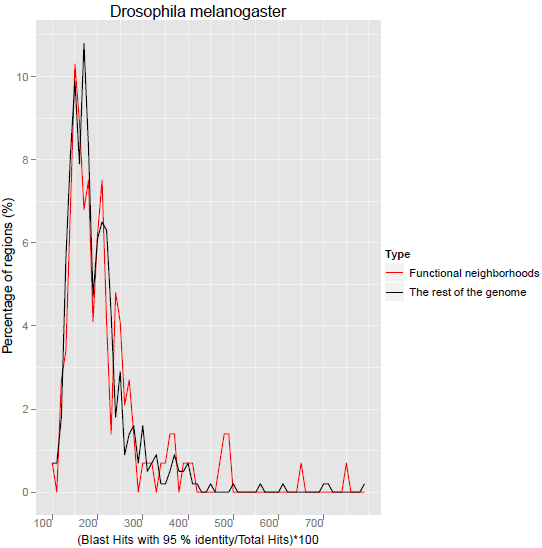


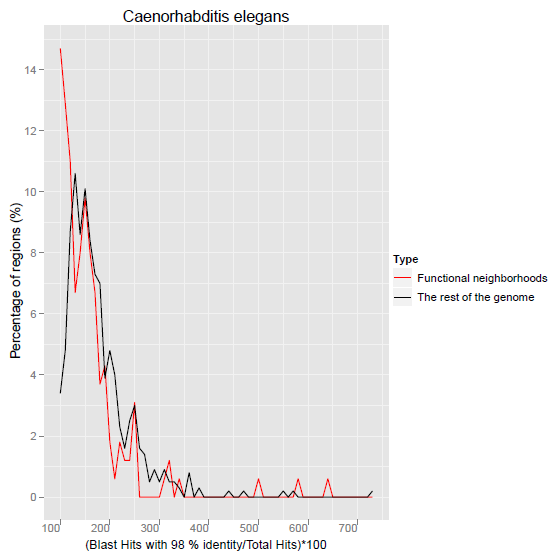


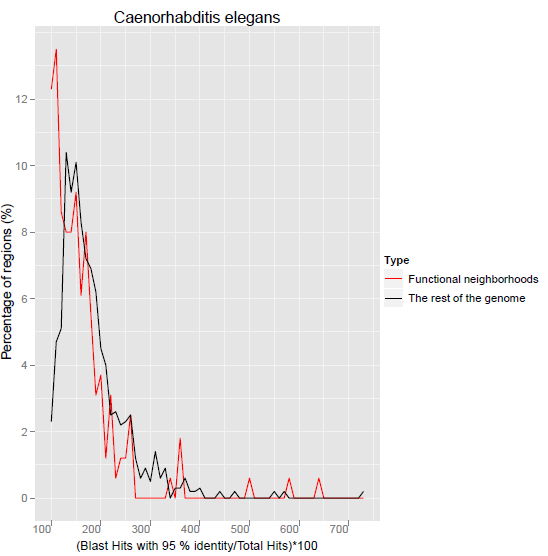


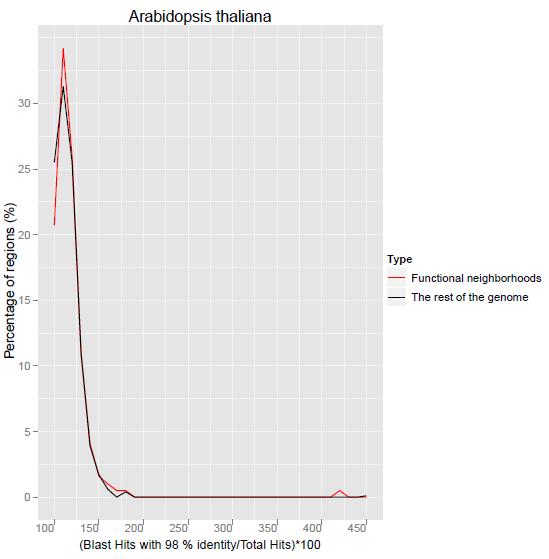


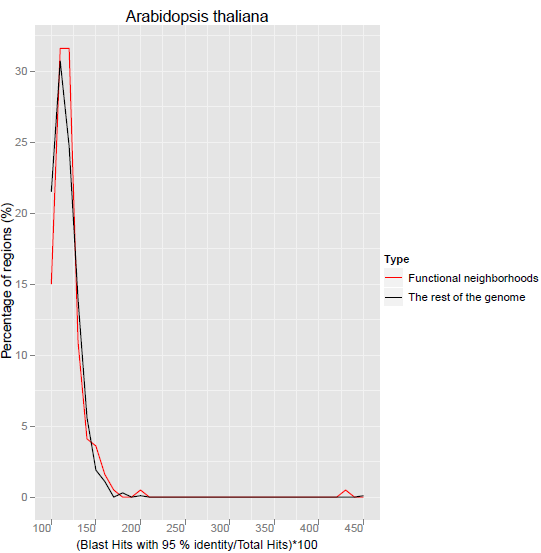

Supplement: Figure S3 — Distribution of BLASTP hits with an identity over the 98% and 95% in the different genomes studied for the functional neighborhoods (red) and for the rest of the genome (black). (0.57 MB DOC) [file pcbi.1000953.s003.doc]
