# Supplementary material for: Selection upon Genome Architecture: Conservation of Functional Neighborhoods with Changing Genes
Source: PLoS Comput Biol. 2010 Oct 7;6(10):e1000953. doi: 10.1371/journal.pcbi.1000953 (PMC2951340; doi:10.1371/journal.pcbi.1000953)
Supplement: Table S1 — Duplication events t-test comparing the distribution of BLASTP hits for functional neighborhoods versus BLASTP hits for the rest of the genome. (0.04 MB DOC) [file pcbi.1000953.s005.doc]

**Supplementary information**

**Selection upon genome architecture: conservation of functional neighborhoods with changing genes**

## Fátima Al-Shahrour, Pablo Minguez, Tomás Marqués-Bonet, Elodie Gazave, Arcadi Navarro and Joaquín Dopazo

**Table S1.** Duplication events t-test comparing the distribution of BLASTP hits for functional neighborhoods versus BLASTP hits for the rest of the genome.

| **Organism** | **95 % BLASTP identity** | **98 % BLASTP identity** |
| --- | --- | --- |
|  | **p-value** | **p-value** |
| *Homo sapiens* | 0.81 | 0.96 |
| *Pan troglodytes* | 0.17 | 0.29 |
| *Mus musculus* | 0.68 | 0.16 |
| *Rattus norvegicus* | 0.11 | 0.22 |
| *Danio rerio* | 0.29 | 0.3 |
| *Caenorhabditis elegans* | 0.003 | 0.004 |
| *Drosophila melanogaster* | 0.03 | 0.06 |
| *Arabidopsis thaliana* | 0.13 | 0.21 |
| *Gallus gallus* | 0.06 | 0.1 |
