# Supplementary material for: Selection upon Genome Architecture: Conservation of Functional Neighborhoods with Changing Genes
Source: PLoS Comput Biol. 2010 Oct 7;6(10):e1000953. doi: 10.1371/journal.pcbi.1000953 (PMC2951340; doi:10.1371/journal.pcbi.1000953)
Supplement: Table S2 — Sliding window sizes used for scanning all the chromosomes of the studied species. Window size was adjusted in each species to contain, on average, approximately 50 genes. (0.04 MB DOC) [file pcbi.1000953.s006.doc]

**Supplementary information**

**Selection upon genome architecture: conservation of functional neighborhoods with changing genes**

## Fátima Al-Shahrour, Pablo Minguez, Tomás Marqués-Bonet, Elodie Gazave, Arcadi Navarro and Joaquín Dopazo

**Table S2.** Sliding window sizes used for scanning all the chromosomes of the studied species. Window size was adjusted in each species to contain, on average, approximately 50 genes.

| **Organism** | **Sliding windows size** | **Average genes per window** |
| --- | --- | --- |
| *Homo sapiens* | 5 Mbps | 54.2 |
| *Pan troglodytes* | 5 Mbps | 52.32 |
| *Mus musculus* | 5 Mbps | 50.16 |
| *Rattus norvegicus* | 5 Mbps | 45.88 |
| *Gallus gallus* | 3,5 Mbps | 54.81 |
| *Danio rerio* | 3,5 Mbps | 54.54 |
| *Drosophila melanogaster* | 400 Kbps | 48.17 |
| *Caenorhabditis elegans* | 250 Kbps | 51.95 |
| *Arabidopsis thaliana* | 200 Kbps | 50.09 |
